# The risks associated with the widespread use of telemedicine in oncology: Four cases and review of the literature

**DOI:** 10.1002/cnr2.1531

**Published:** 2021-08-18

**Authors:** François Panet, Marianne Tétreault‐Langlois, Vincent Morin, Khalil Sultanem, David Melnychuk, Lawrence Panasci

**Affiliations:** ^1^ Department of Oncology McGill University, Jewish General Hospital Montréal Québec Canada; ^2^ Department of Medicine, Faculty of Medicine University of Sherbrooke Sherbrooke Quebec Canada

**Keywords:** COVID‐19, oncology, telemedicine

## Abstract

**Background:**

COVID‐19 changed the way we practice oncology in multiple ways. Because most cancer patients are comorbid or immunocompromised, we are trying as much as possible to reduce their risk of infection. Marginal just 2 years ago, telemedicine quickly became preeminent with the pandemic to reduce hospital exposure. However, using only virtual visits in oncology patients risk delaying cancer diagnosis or the identification of a complication.

**Case series:**

We present here four cases where a serious medical problem evident on physical exam was overlooked during a virtual visit. Two of our patients experienced a delay in cancer diagnosis thus putting them at risk of local or distant spread. The two others were established oncology patients where a serious medical complication was missed on a virtual visit.

**Conclusions:**

Now more than a year into the pandemic, telemedicine has clearly been a useful tool by limiting unnecessary hospital visits. Yet, as our cases illustrate, its use in oncology without clear boundary can undermine the quality of care. Now that effective vaccines are reducing the transmission and the severity of infection, most oncology patients can be evaluated by a real‐time visit.

## 1 INTRODUCTION

The COVID‐19 pandemic changed the way we practice medicine in multiple ways. To limit the spread of the virus, we try to avoid unnecessary visits to the hospital. Recently, there have been numerous articles published on telemedicine as a way to limit contact to the healthcare system.^1^, ^2^ This is an interesting option in oncology since many of our patients are immunocompromised and could be at an increased risk of COVID‐19 infection. However, they are also at high risk of complications from their cancer or the therapy administered and require frequent medical follow up. We think that patients, who are frail, present worrisome symptoms, or who are on active chemotherapy and/or immunotherapy treatments are better served with real‐time visits. To illustrate this, we recently treated four patients who had serious medical problems that were either missed on virtual follow‐ups or the patient had not had a visit after a serious problem. These cases illustrate the risk associated with the widespread use of telemedicine in oncology.

### 1.1 Case #1

A 67‐year‐old healthy female presented with a history of mild dysphagia and a white spot seen on the right tonsillar fossa for 3–4 weeks. She consulted her family doctor and had a virtual visit with the recommendation of conservative management. Due to persistent symptoms, the patient made an appointment with an Ear Nose and Throat (ENT) specialist. She had a virtual appointment with the ENT specialist in March 2020 during which the doctor requested that she take a picture of her throat with the mobile camera and email it to him. The ENT specialist reassured her and recommended water and salt rinses. Three months passed with persistent and progressive symptoms. The husband insisted on an on‐site consultation with ENT. The on‐site consult took place 3 months later. The examination revealed a suspicious right tonsillar mass and right level II cervical lymphadenopathy. The patient ultimately was diagnosed with stage cT2N1 squamous cell carcinoma of the tonsil and underwent combined modality chemo‐radiation. If diagnosed earlier, the patient may have been able to be treated with a single modality therapy such as robotic surgical resection or limited volume radiation.

### 1.2 Case #2

A 68 year old ex‐smoker male with no significant past medical history presented with a 2 month history of a persistent sensation of a foreign body in his throat, mild dysphagia, and a painless left neck 2 cm mass. He was assessed by his family doctor who requested a consult with an ENT specialist. The patient had a virtual consultation with an ENT specialist in April 2020. The history was obtained and the patient was given a prescription for an antibiotic and a rinse with recommendation of follow‐up if his symptoms persisted. The patient was reassured and continued on the prescribed remedies for 6–8 weeks. Due to progressive dysphagia, the patient requests a follow‐up visit, which was done virtually. Two weeks after in July 2020, the patient was seen in person and on examination, a large base of the tongue mass with bilateral cervical lymphadenopathies was noted. The patient was diagnosed with stage cT3N2M0 squamous cell carcinoma P16 negative and underwent combined chemo‐radiation. If diagnosed earlier, the patient may have been able to be treated with a single modality therapy such as robotic surgical resection or limited volume radiation.

### 1.3 Case #3

A 77‐year‐old female with a history of triple negative breast cancer resected in 2004 presented with fatigue in our clinic for a routine follow‐up visit. Approximately 4 months prior to her real time visit with us, she had been hospitalized for congestive heart failure secondary to malfunction of her aortic prosthetic valve. She was scheduled to have a follow‐up virtual visit with her treating cardiologist. During the visit with us, she admitted on questioning that she had gained weight (45 pounds). Her physical examination revealed anasarca and the presence of cardiac murmurs. An echocardiogram showed aortic valve dysfunction and severe tricuspid valve insufficiency. She was admitted for treatment of severe congestive heart failure.

### 1.4 Case #4

A 67‐year‐old patient who has metastatic breast cancer received weekly paclitaxel chemotherapy for 2 months. She was also known for congestive heart failure and renal insufficiency. She had not seen an MD in real‐time since the start of her chemotherapy in August 2020. Despite diligent virtual follow‐ups, the patient developed florid congestive heart failure, which was missed by virtual visits. It was a chemotherapy unit nurse who requested the patient be seen by a doctor. She was hospitalized in cardiology for more than a month after an examination that showed evidence of congestive heart failure.

## 2 DISCUSSION

These four examples illustrate the drawbacks of telemedicine, while highlighting the importance of in‐person medical visits to discover important findings on physical exam. The widespread use of virtual medicine has been readily adopted in part because we rely more and more on technology rather than the physical examination. In 50 years, new technologies such as ultrasonography, CT scans, magnetic resonance imaging and PET scans have been introduced. These new technologies have greatly aided physician in diagnosing and following serious medical illnesses such as cancer. However, with the use of more complex chemotherapies and immunotherapies, there is still a need to examine patients, particularly to detect cardiac arrhythmias, signs of cardiac failure or pulmonary toxicities associated with such therapies. There are attempts to overcome those telehealth limitations, for example by making the patient take his own vital signs or using technology to analyze his heart rhythm.^3^ However, virtual physical exams are not standardized and, in our experience, commonly omitted during a telehealth appointment. We therefore use telemedicine appointments only in patients not requiring to be examined, for example those on surveillance imaging. For patients on active anti‐cancer therapy or presenting symptoms we preferably use real‐time visits. In the future, new tools could improve the reliability of virtual physical exam.

Furthermore, we are using telemedicine to reduce the risk of contracting COVID‐19 for healthcare workers and patients, but with adequate protection, the risk seems low. One trial documented that, with adequate PPE, nosocomial transmission of COVID‐19 in hospitalized patients is as low as 0.025%.^4^ Likewise, with frequent testing, the rate of COVID‐19 infection in a tertiary cancer center in Australia was similar to the general population, even if the majority of patients were on active treatment and had frequent hospital visits.^5^ In Singapore, the National Cancer Center kept in person clinic appointment for patients on active anti‐cancer therapy without any outbreak of covid‐19.^6^ Also, at our institution, we are noticing that virtual visits can increase the workload of those who work in real‐time. For example, the nurses on the chemotherapy unit are now finding clinically relevant problems missed by virtual visits. Additionally, we now have evidence that vaccines provide adequate immunity after two doses in cancer patients further reducing the risk associated with hospital exposure.^7^ Thus, with adequate protection, frequent testing and vaccines, we can safely evaluate selected patients with real‐time visits to offer optimal care.

Moreover, while the preliminary studies suggested worst outcomes in cancer patients with COVID‐19, these studies had flaws, overestimating the actual risk in our population.^8^ Newer reports indicate that neither cancer nor chemotherapy are risk factors for severe COVID‐19 infection.^9^, ^10^ Another meta‐analysis found a moderate increase in death from infection in patients with malignancy, with a relative risk of 1.66 (*p* < .0001) compared to the general population, but not in those over 65 years old.^11^ Still, the risk of severe COVID‐19 seems particularly higher in hematological malignancy, especially multiple myeloma. Since neutropenia can be associated with a greater risk of COVID‐19 complications,^8^ we altered our practice to utilize granulocyte colony stimulating factors more liberally, even with moderately myelosuppressive chemotherapy.

In addition, without clear boundaries, telemedicine can cause collateral damage. Because residents and medical students learn best by seeing patients, we are concerned that telemedicine may be affecting their clinical exposure. It is tantamount to preserve medical education unless there are overwhelming safety issues.

Besides, no study has evaluated the feasibility and safety of telemedicine oncology follow up in underserved populations like the elderly or immigrants. Indeed, on virtual follow‐ups we have noticed that these patients tend to be less capable of verbalizing their concerns. This is often related to the telemedicine technology utilized or language barriers.

The COVID‐19 pandemic is undeniably bringing new challenges in oncology and patient care remains our top priority. Fortunately, recent evidence indicates that with adequate measures we can safely evaluate selected patients with real‐time oncology visits. Furthermore, even if the actual risk of COVID‐19 infection in most cancer patients remains controversial, recent reports reveal that the outcome is not much different from that of the general population except in high‐risk groups such as patients with hematologic malignancies or on significantly myelosuppressive chemotherapy. Effective vaccines now reduce this risk further. Telemedicine has proved to be a useful tool to follow patients in certain situations, but as our cases illustrate its widespread use in oncology involves risks. We recommend that such risks be weighed against COVID‐19 infection risk in our patients to provide the best possible care.

## CONFLICT OF INTEREST

The authors have stated explicitly that there are no conflicts of interest in connection with this article.

## AUTHORS' CONTRIBUTIONS

All authors had full access to the data in the study and take responsibility for the integrity of the data and the accuracy of the data analysis. *Conceptualization*, F.P., V.M., K.S.; *Data Curation*, F.P., V.M., K.S., L.P.; *Writing ‐ Original Draft*, L.P.; *Writing ‐ Review & Editing*, F.P., V.M., M.T.‐L., D.M., K.S., L.P.

## ETHICAL STATEMENT

This case series was completed in agreement with institutional ethics regulation.

## Data Availability

Data sharing not applicable to this article as no datasets were generated or analysed during the current study.
